# Imbalanced Hemolymph Lipid Levels Affect Feeding Motivation in the Two-Spotted Cricket, *Gryllus bimaculatus*

**DOI:** 10.1371/journal.pone.0154841

**Published:** 2016-05-04

**Authors:** Takahiro Konuma, Yusuke Tsukamoto, Hiromichi Nagasawa, Shinji Nagata

**Affiliations:** 1 Department of Applied Biological Chemistry, Graduate School of Agricultural and Life Sciences, the University of Tokyo, Tokyo, Japan; 2 Department of Integrated Biosciences, Graduate School of Frontier Sciences, the University of Tokyo, Chiba, Japan; University of Würzburg, GERMANY

## Abstract

Insect feeding behavior is regulated by many intrinsic factors, including hemolymph nutrient levels. Adipokinetic hormone (AKH) is a peptide factor that modulates hemolymph nutrient levels and regulates the nutritional state of insects by triggering the transfer of lipids into the hemolymph. We recently demonstrated that RNA interference (RNAi)-mediated knockdown of the AKH receptor (AKHR) reduces hemolymph lipid levels, causing an increase in the feeding frequency of the two-spotted cricket, *Gryllus bimaculatus*. This result indicated that reduced hemolymph lipid levels might motivate crickets to feed. In the present study, to elucidate whether hemolymph lipid levels contribute to insect feeding behavior, we attempted to manipulate hemolymph lipid levels via the lipophorin (Lp)-mediated lipid transferring system in *G*. *bimaculatus*. Of the constituent proteins in Lp, we focused on apolipophorin-III (GrybiApoLp-III) because of its possible role in facilitating lipid mobilization. First, we used RNAi to reduce the expression of GrybiApoLp-III. RNAi-mediated knockdown of *GrybiApoLp-III* had little effect on basal hemolymph lipid levels and the amount of food intake. In addition, hemolymph lipid levels remained static even after injecting AKH into *GrybiApoLp-III*^RNAi^ crickets. These observations indicated that ApoLp-III does not maintain basal hemolymph lipid levels in crickets fed *ad libitum*, but is necessary for mobilizing lipid transfer into the hemolymph following AKH stimulation. Second, Lp (containing lipids) was injected into the hemolymph to induce a temporary increase in hemolymph lipid levels. Consequently, the initiation of feeding was delayed in a dose-dependent manner, indicating that increased hemolymph lipid levels reduced the motivation to feed. Taken together, these data validate the importance of basal hemolymph lipid levels in the control of energy homeostasis and for regulating feeding behavior in crickets.

## Introduction

The feeding behavior of insects has been extensively investigated, particularly in the migratory locust, *Locusta migratoria* [1–3]. The feeding behaviors of *L*. *migratoria* occur at regular intervals [2], and they are assumed to result from a repertoire of physiologically important causal factors [3, 4]. For example, fullness of the hindgut [5], dietary nutrients [6], nutrient content in the hemolymph [7], and light stimulation [8] have been demonstrated to display feeding rhythmicity in this species. Although the physiological relationship between feeding behavior and these causal factors has been investigated, limited information is available at the molecular level.

Our research group has focused on how the nutrient content in the hemolymph affects feeding behavior in crickets at the molecular level. Hemolymph nutrient levels are influenced by the equilibration between the utilization of stored nutrients and ingested nutrients/metabolites [9]. In insects, the fat body functionally corresponds to adipocytes and the liver of vertebrates [10, 11]. The fat body stores metabolic fuels, such as glycogen and triacylglycerol, and synthesizes the hemolymph sugar, trehalose [12]. Consequently, hemolymph nutrient and metabolite levels might be indicators for maintaining nutrient levels, possibly reflecting the demand for nutrients.

Among several essential nutrients, the levels of hemolymph sugars and lipids are modulated by adipokinetic hormone (AKH), which is thought to be functionally related to mammalian glucagon [13]. AKH is a peptide hormone, originally identified as a stimulating factor for lipid mobilization and locomotor activity in locusts [14, 15]. AKH is a highly conserved peptide hormone in insects, and AKHs have been identified from over 40 insect species [14].

Water-insoluble nutrients in hemolymph, including lipids, require the presence of carrier or scavenger proteins to maintain optimal levels and to enable transport between organs. The Lipophorin (Lp) family of plasma lipoproteins participates in lipid transport during the mobilization process of lipid entry into hemolymph by acting as reusable shuttle particles [16, 17]. High-density Lp (HDLp) is the main lipophorin in the hemolymph of resting insects. HDLp is generally composed of two apolipoproteins: ApoLp-I (250,000 Da) and ApoLp-II (80,000 Da) [18]. Another predominant apolipoprotein of Lp, ApoLp-III (18,000 Da) is found in the hemolymph as its free form and as a complex associated with HDLp [19, 20]. This complex form, called low-density Lp (LDLp), is formed when HDLp is required for loading additional lipids, such as diacylglycerol (DAG), which is a hydrolyzed lipid of triacylglycerol (TAG) located in the AKH-stimulated fat body [19, 20]. It is implied that AKH is the key peptide hormone necessary to maintain lipid homeostasis in insects, including the maintenance of hemolymph lipid levels and mobilization of lipids from the fat body into hemolymph.

In addition to its energy-mobilizing activity, AKH influences the regulation of feeding-related behavior, including starvation-driven hyperlocomotion (e.g., food-searching behavior) [21, 22]. We previously showed that nutrients, particularly lipids controlled by AKH signaling, may affect the feeding behavior of the two-spotted cricket *Gryllus bimaculatus* [23]. Knockdown of the AKH receptor in *G*. *bimaculatus* (*GrybiAKHR*) by RNA interference (RNAi) reduced the lipid and carbohydrate levels in the hemolymph, which significantly increased feeding frequency [23]; however, the mechanism of how reductions in lipids and carbohydrates lowered hemolymph nutrient levels to affect feeding behavior at the molecular level has yet to be elucidated.

Because imbalanced lipid levels in *GrybiAKHR*^RNAi^ crickets cause changes in feeding frequency [23], we focused on hemolymph lipid levels as a possible causal factor of feeding behavior in crickets. To determine whether hemolymph lipids contribute to insect feeding behavior, we sought to modulate hemolymph lipid levels by two experimental parameters: (i) transcriptional manipulation through RNAi targeting *ApoLp-III* in *G*. *bimaculatus* (*GrybiApoLp-III*), and (ii) injection of *G*. *bimaculatus* Lp (GrybiLp) into the hemolymph. Finally, using data from these experiments, we discuss how hemolymph lipid mobilization facilitated by Lp potentially affects hemolymph lipid levels, and whether changes in hemolymph lipid levels affect the feeding behavior of two-spotted crickets.

## Materials and Methods

### Insects

Fifth instar larvae of the two-spotted cricket *G*. *bimaculatus* were purchased from Tsukiyono farm Co., Ltd. (Gunma, Japan). Crickets were reared in plastic containers (55 × 39 × 31 cm) at 27 ± 1°C, 70 ± 5% relative humidity, and under long-day lighting conditions (16L: 8D). Crickets were fed *ad libitum* on a standard diet for rabbits (ORC4; Oriental Yeast Co., Ltd., Tokyo, Japan) with unlimited access to water. Although most of the crickets molted simultaneously, we selected crickets with synchronous growth from the fifth instar to adult emergence. In all experimental procedures, excluding the feeding assay, we used adult male crickets on the day they emerged. These crickets were kept isolated in plastic containers (17 × 10 × 12 cm) until they were used in experiments.

### Identification of GrybiApoLp-III

Hemolymph collected from crickets was subjected to SDS-PAGE. Hemolymph was collected by pipetting using a micropipette adjusted to 5 μL. The collected hemolymph was immediately transferred into ice-cold loading buffer to prevent clotting before SDS-PAGE. The gel bands stained with Coomassie Brilliant Blue (CBB) were excised. Individual gel pieces were submerged in methanol for 10 min to dehydrate. The resulting gel pieces were dried and then trypsinized at 37°C for 14 h by swelling with the 0.1% sequence grade trypsin (Roche) in 0.1 M ammonium acetate. The resulting peptide fragments were extracted from the gel piece by two submersions in 60% acetonitrile containing 0.1% TFA. The collected peptide fragments were subjected to RP-HPLC with a PEGASIL 300 ODS column (4.6 i.d. × 250 mm) using a linear gradient program (10–60% acetonitrile in 0.05% TFA over 40 minutes at a flow rate of 1 mL/min). Peaks were detected at an absorbance of 225 nm. Four significant peaks were recovered and were subjected to amino acid sequencing using a Procise cLC protein sequencer (Applied Biosystems, Foster City, CA).

### Native-PAGE analysis of GrybiApoLp-III

To analyze the hemolymph to determine the free GrybiApoLp-III level, the collected hemolymph (2.2 μL) was subjected to native-PAGE stained with CBB. Hemolymph was collected by pipetting and was then transferred into an ice-cold tube using a micropipette as described above. During native-PAGE analysis, a band corresponding to free GrybiApoLp-III was confirmed by N-terminal amino acid sequence analysis on a Procise cLC protein sequencer (data not shown). The quantity of free GrybiApoLp-III was evaluated by comparing its density with that of the CBB-stained bands.

### cDNA cloning of putative *GrybiApoLp-III*

Crickets were anesthetized at 4°C and the fat body was dissected out. The resulting total RNAs from the tissues were extracted using TRIZOL reagent (Invitrogen, Carlsbad, CA) according to the manufacturer’s protocol. Resulting total RNAs were treated with RQ DNase I (Promega, Madison, WI). The extracted total RNAs (500 ng each) were reverse-transcribed using Superscript III RNaseH^-^reverse transcriptase (Invitrogen) and an oligo (dT) primer [5′-AAGGAGTGGTATCCAGTGTGCTGG(T)_30_VN-3′]. The PCR (polymerase chain reaction) targeting a *GrybiApoLp-III* partial cDNA fragment was performed using a set of specific primers that were designed based on the nucleotide sequences of *Acheta domesticus ApoLp-III* [20]; AchdoApoLp-III-F, 5′-ACCATCCAGAACGCGCTGCCT-3′ and AchdoApoLp-III-R, 5′-GCACAGACTGCTGCACCTGGTT-3′. The PCR was performed using a GeneAmp PCR System 9700 (Applied Biosystems, Foster City, CA) with TaKaRa Ex Taq polymerase (TaKaRa Bio, Shiga, Japan) at the following PCR conditions: an initial denaturation step at 94°C for 3 min, followed by 35 cycles of amplification (94°C for 30 sec, 50°C for 30 sec, 72°C for 1 min for the first PCR and nested PCR). All PCR runs were performed under the same conditions as those described above except for changes to the annealing temperature and extension time. 3′-RACE (rapid amplification of cDNA end) was performed using a FirstChoice RLM-RACE Kit (Ambion Inc., Austin, TX). For 3′-RACE of *GrybiApoLp-III*, the 3′-RACE outer primer and ApoLp-III-3R1 (5′-ACCATCCAGAACGCGCTGCCTT-3′) were used for the first round of PCR with 35 amplification cycles (94°C for 30 sec, 60°C, for 30 sec, 72°C for 2 min). The 3′-RACE inner primer and ApoLp-III-3R2 (5′-AGGAAGTGCGCACGCA-3′) were used for nested PCR with 35 amplification cycles (94°C for 30 sec, 50°C for 30 sec, 72°C for 2 min). PCR products were electrophoresed and extracted using a QIAquick Gel Extraction Kit (Qiagen, CA), which were then subcloned into a pGEM-T vector (Promega) using a cDNA Ligation Kit Ver. 2.1 (TaKaRa Bio) and transformed into XL1-Blue *Escherichia coli*. The inserted cDNAs were sequenced using a BigDye Terminator v3.1 Cycle Sequencing Kit (Applied Biosystems) on an ABI PRISM 310 Genetic Analyzer (Applied Biosystems). The accession number of Genbank for GrybiApoLp-III is AHF53422.1.

### Sequence alignment and phylogenetic analysis

The deduced amino acid sequence of GrybiApoLp-III was aligned with those of known and annotated ApoLp-IIIs, which were obtained from GenBank using BLAST (Basic Local Alignment Search Tool) searches (blastn and tblastn programs). Amino acid sequences of ApoLp-IIIs were aligned using ClustalW. A phylogenetic tree was generated by the Neighbor-joining method. ApoLp-III sequences used in the phylogenetic analysis included (GenBank accession number following species abbreviation in parenthesis): *Gryllus bimaculatus* (Grybi), KC684976; *Spodoptera exigua* (Spoex), AEW24424; *Nilaparvata lugens* (Nillu), ADE34171; *Trichoplusia ni* (Trini), ABV68867; *Hyphantria cunea* (Hypcu), AAQ24031; *Plutella xylostella* (Pluxy), ADK78218; *Manduca sexta* (Manse), AAA29300; *Galleria mellonella* (Galme), CAA07363; *Bombyx mori* (Bommo), NP_001037078; *Bombyx mandarina* (Bomma), AAB02851; *Aedes aegypti* (Aedae), XP_001659524; *Culex quinquefasciatus* (Culqu), EDS29975; *Anopheles gambiae* (Anoga), ADM86753; *Anopheles sinensis* (Anosi), ADN52300; *Acheta domesticus* (Achdo), AAA64737; *Tribolium castaneum* (Trica), EFA05722; *Locusta migratoria* (Locmi), AAA29282; and *Homo sapiens* (Homsa) apolipoprotein-AI (an outgroup), CAA30377.

### Expression analysis using RT-PCR (reverse-transcription-polymerase chain reaction)

Total RNA extraction and subsequent cDNA synthesis was performed as described above. Tissues (fat body, foregut, midgut, hindgut, Malpighian tubule, trachea, muscle, ovary, testis, and nervous system) were carefully dissected from anesthetized crickets. Hemocytes were collected after separation from hemolymph by centrifugation for 3 min at 2,000 × *g* at 4°C. Partial cDNA fragments of *GrybiApoLp-III* and elongation factor-1α (*GrybiEF*) (GenBank accession number: AB583234.1) were amplified using the following primers: ApoLp-III-Fw (5′-AGGAGGAAGTGCGCACGCAA-3′); ApoLp-III-Rv (5′-GCAGTCTTGAGGGACTCTGCGA-3′); EF-Fw (5′-ATGCCTGTATCTTGACTGCTCA-3′); and EF-Rv (5′-ATGGTTTGCTTCCAGTTTCAGT-3′). PCR conditions were as follows: 94°C for 30 sec, 50°C for 30 sec, 72°C for 1 min for 31 cycles for *GrybiApoLp-III* and 30 cycles for *GrybiEF*.

### RNA interference

To prepare dsRNA, template cDNA was generated by PCR using primers targeting fragments of *GrybiApoLp-III* and *EGFP* (enhanced green fluorescent protein) cDNA with an additional T7 promoter sequence (underlined) at the 5′-terminal end: T7-ApoLp-III-Fw (5′-GCTTCTAATACGACTCACTATAGCAGACCTTTGCCAACAACGT-3′); T7-ApoLp-III-Rv (5′-GCTTCTAATACGACTCACTATAGACAGACTGCTGCACCTCCTT-3′);, T7-EGFP-Fw (5′-GCTTCTAATACGACTCACTATAGAGCTGACCCTGAAGTTCATCTG-3′); and T7-EGFP-Rv (5′-GCTTCTAATACGACTCACTATAGCTTGTACAGCTCGTCCATGC-3′). RNAs were synthesized with T7 RNA polymerase using 500 ng of the PCR products as template DNAs. The synthesized RNAs were purified by phenol/chloroform extraction and ethanol precipitation, and then dissolved in diethylpyrocarbonate (DEPC)-treated water to a final concentration of 3 μg/μL. Complementary RNAs were denatured for 5 min at 100°C and cooled to room temperature for annealing overnight. For transcriptional knockdown, the prepared dsRNA solutions (*GrybiAKHR*-dsRNA and *EGFP*-dsRNA) (3 μg and 1 μL, respectively) were administered orally [23] on the day of adult emergence for transcriptional knockdown. Non-target effects of EGFP-dsRNA treatment were confirmed by using different dsRNA encoding DsRed II. The dsRNA was prepared using following primers: T7-DsRed2Fw (5′-GCTTCTAATACGACTCACTATAGAGAACGTCATCACCGAGTTCAT-3’) and T7-DsRed2rev (5′-GCTTCTAATACGACTCACTATAGCCGATGAACTTCACCTTGTAGA-3′) with pDsRedII as template DNA. Efficiency of RNAi was evaluated by quantitative RT-PCR using a Thermal Cycler Dice Real Time System (TaKaRa Bio). The reaction was carried out using SYBR-premix Ex taq-II (TaKaRa Bio) with the specific primers 5′-TCGCAGAGTCCCTCAAGACT-3′ and 5′-AGACTGCTGCACCTGGTTG-3′. To evaluate the reaction using the ΔCt method, we used primers specific to a house-keeping gene, Elongation Factor (5′-CCCTGCTGCTGTTGCTTT-3′ and 5′-CCCATTTTGTCGGAGTGC-3′).

### Lipid and carbohydrate extraction from hemolymph and the fat body

Lipids were extracted using established protocols [23, 24]. In brief, cricket hemolymph (5 μL) was collected into a tube containing 10 mg sodium sulfate and 100 μL 75% methanol, which was then homogenized in 300 μL of chloroform/methanol (1:1 ratio), vortexed, and centrifuged (15,300 × *g* at 4°C for 10 min). The supernatant was transferred to a new tube and then vortexed and centrifuged (15,300 × *g* at 4°C for 10 min) following the addition of 150 μL chloroform and 250 μL 1 M NaCl in distilled water. The organic lipid-containing layer was dried under vacuum centrifugation. The resulting oily liquid was used for quantification of lipids.

### Lipid quantification

Extracted hemolymph lipids were measured using the sulfo-phospho-vanillin method [23, 25]. Extracted lipids in a 1:1 ratio of chloroform/methanol (1 μL) mixed with 50 μL of sulfuric acid were heated at 100°C for 10 min. After cooling, 500 μL of vanillin reagent (0.2% vanillin in 67% ortho-phosphoric acid) was added. The resulting samples were measured at 540 nm. Cholesterol (Sigma-Aldrich, Tokyo, Japan) was used as a standard lipid. The resulting amount of hemolymph lipids were quantified as that of DAG.

### Preparation and injection of GrybiAKH

GrybiAKH (pQVNFSTGWamide) was synthesized and purified as reported previously [23]. GrybiAKH was injected into the hemolymph through the abdominal cavity with the needle oriented towards the head. Five pmol of GrybiAKH in 3 μL water, or 3 μL Ringer’s solution [140 mM NaCl, 4.7 mM KCl, 2.0 mM CaCl_2_, 2.0 mM MgCl_2_, 5 mM HEPES-NaOH (pH 7.1)] was injected into each cricket. To measure hemolymph lipids, 5 μL of hemolymph was collected immediately prior to injection and again 90 min after injection.

### Food intake assay

On the day of adult emergence, virgin female and male crickets were treated with 3 μg *GrybiApoLp-III*-dsRNA or *EGFP*-dsRNA and then were kept isolated in plastic containers (17 × 10 × 12 cm). The approximate amount of food intake was evaluated according to an index of food intake as defined previously [23] by counting the number of fecal pellets every 24 hours for ten days.

### Collection and injection of Lp from hemolymph

Hemolymph was collected directly into an ice-cold tube. Collected hemolymph (700 μL) was centrifuged (1,000 × *g* for 10 min) to remove hemocytes. The supernatant was mixed with 1 mL phosphate buffered saline (PBS). Using this solution, GrybiLp was isolated by KBr gradient ultracentrifugation (40,000 × *g* for 16 h) (Optima^TM^ L-70R Ultracentrifuge; Beckman Coulter, Brea, CA). Following ultracentrifugation, each fraction was weighed to calculate its density. Each fraction was desalted against PBS and concentrated to 50 μl by passing through an Ultrafree-MC 10,000 NMWL Filter Unit (Amicon Millipore, Bedford, MA). The amount of lipid in each fraction was measured after lipid extraction as described above. These lipid fractions containing Lp were used in the experiments. One μl of the Lp-dissolved solution was injected into each cricket.

### Statistical analyses

Comparison of the two experimental groups was performed using the Mann–Whitney test (Fig 6D). Multiple comparisons were performed using Tukey’s HSD test (Fig 4) and Dunnett’s test (Fig 6C). *P*-values less than 0.05 were considered statistically significant. All experiments were repeated for confirmation of reproducibility. In all figures, the data from the representative and reproducible results among the trials of the same sized experiments are described.

## Results

### Identification of *Gryllus bimaculatus* ApoLp-III

**Fig 1:**
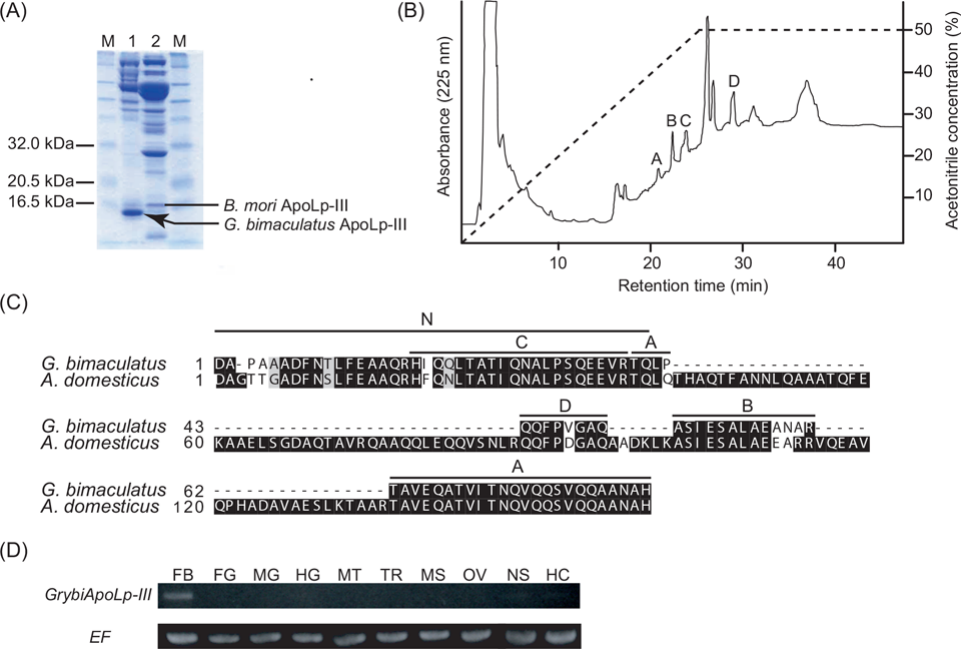
Identification of GrybiApoLp-III. (A) Analysis of hemolymph proteins from *G*. *bimaculatus* (lane 1) and *B*. *mori* (lane 2) separated by SDS-PAGE. *B*. *mori* ApoLp-III is indicated by a bar. The candidate for GrybiApoLp-III is indicated by an arrow. (B) The RP-HPLC profile of gel-digested proteins from the band corresponding to the GrybiApoLp-III candidate. Peaks A–D were subsequently subjected to amino acid sequence analyses. (C) Alignment of amino acid sequences of the resulting GrybiApoLp-III and *A*. *domesticus* ApoLp-III sequences. Bars above sequences indicate the results of amino acid sequence analyses. N-terminal sequence, from 1st residue (D) to 38th residue (L), and fragment sequences from peaks A–D in RP-HPLC indicate N and A–D, respectively. (D) Tissue distribution of *G*. *bimaculatus ApoLp-III* by RT-PCR. Elongation factor (*EF*) was used as an experimental control. FB, Fat body; FG, foregut; MG, midgut; HG, hindgut; MT, Malpighian tubules; TR, trachea; MS, muscle; OV, ovary; NS, nervous system; HC, hemocytes.

Because ApoLp-III in *G*. *bimaculatus* (GrybiApoLp-III) has not yet been identified, we performed an SDS-PAGE analysis to identify that protein using *G*. *bimaculatus* hemolymph (Fig 1A). We observed a predominant band similar in size to ApoLp-III from the hemolymph of *Bombyx mori* [26] (Fig 1A). Sequencing of the protein’s N-terminus and trypsinized peptides (Fig 1B) revealed a partial protein sequence that was highly homologous to ApoLp-III in the closely related cricket species, *Acheta domesticus* [27] (Fig 1C). To obtain the entire amino acid sequence of GrybiApoLp-III, we performed RT-PCR using specific primers designed to the identified amino acid sequences and to *A*. *domesticus ApoLp-III*. The RT-PCR analysis revealed a partial sequence encoding *GrybiApoLp-III* (386 bp) within the identified amino acid sequence. The remaining 3′ end of *GrybiApoLp-III* was obtained using the 3′-RACE procedure (S1A Fig). Together with the amino acid sequence analyses, we determined the entire 148 amino acid sequence of the mature GrybiApoLp-III protein (S1B Fig).

The identified amino acid sequence of GrybiApoLp-III exhibited the highest similarity (97%) with that of *A*. *domesticus* ApoLp-III, clustering in the same phylogenetic clade as other insect ApoLp-III proteins (S1C Fig). The RT-PCR analysis, using total RNA extracted from the tissues of adult female and male crickets, confirmed that *GrybiApoLp-III* is predominantly expressed in the fat body, the site of apolipophorin production in other insects [27] (Fig 1D).

### Role of GrybiApoLp-III in rapid lipid mobilization triggered by AKH and long-term starvation

**Fig 2:**
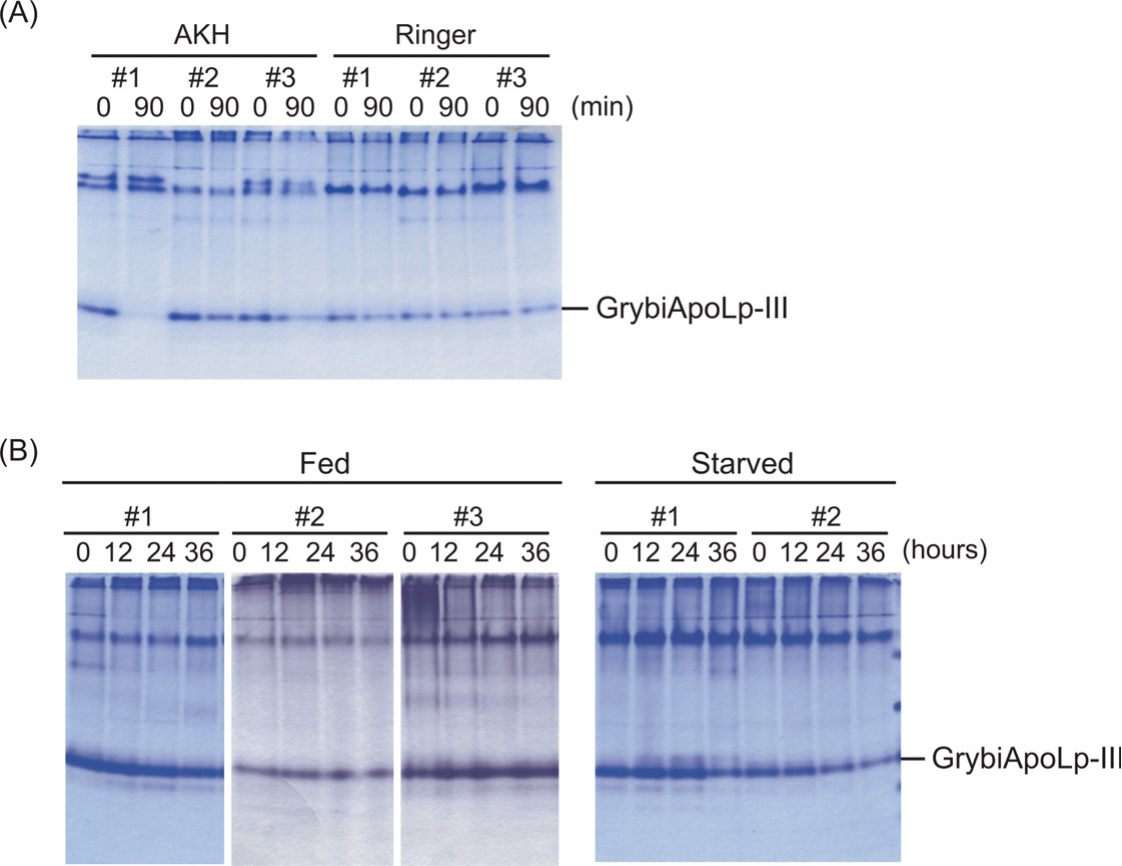
Changes in the amount of free GrybiApoLp-III by AKH stimulation and starvation. (A) Representative data from native PAGE analyses of proteins in *G*. *bimaculatus* hemolymph before and after AKH injection. Free GrybiApoLp-III is indicated by a bar. Figures are representative data from experiments using three individual crickets [the lane number (#1, #2, and #3) indicates the sample from an individual cricket]. Reproducibility of this experiment was confirmed by different experimental trials using more than 30 individuals. (B) Representative data from native PAGE analyses of proteins in hemolymph of starved *G*. *bimaculatus*. Free GrybiApoLp-III is indicated by a bar. Numbers represent individual crickets (#1, #2 and #3). Data on the left (samples from fed crickets) is composed of three single gels. The reproducibility of this experiment was also confirmed by different trials using totally more than 30 individual crickets from different populations.

As AKH is a peptide hormone facilitating the mobilization of lipids from the fat body to hemolymph, we investigated the contribution of GrybiApoLp-III to AKH-stimulated lipid mobilization. To confirm whether GrybiApoLp-III is involved in AKH-induced lipid mobilization from the fat body into hemolymph, we analyzed the proteins in the hemolymph collected before and after AKH injection by native-PAGE analysis (Fig 2A). Because the band corresponding to free GrybiApoLp-III resolved as a separate band from the Lp complex (Fig 2), we were able to visually track the AKH-dependent change in the amount of free GrybiApoLp-III in the hemolymph. AKH injection decreased free GrybiApoLp-III in the hemolymph after 90 min when hemolymph carbohydrate and lipid levels had increased, whereas no change in free GrybiApoLp-III was observed after injecting only Ringer’s solution (Fig 2A). This result suggests that formation of the LDLp complex from HDLp and apoLp-III is required for DAG mobilization, and that GrybiApoLp-III is involved in lipid transfer from the fat body to hemolymph following AKH stimulation in crickets.

Next, we analyzed whether free GrybiApoLp-III in the hemolymph is also affected by starvation. Our native-PAGE analyses showed that starvation for longer than 24 h decreased free levels of GrybiApoLp-III in the hemolymph, whereas free GrybiApoLp-III in crickets fed *ad libitum* remained static (Fig 2B). This decline in free GrybiApoLp-III levels during long-term starvation indicated that free GrybiApoLp-III associates with HDLp to mobilize lipids into the hemolymph, similar to what is observed during AKH-stimulation.

### Confirmation of the effects of RNAi targeting *GrybiApoLp-III*

**Fig 3:**
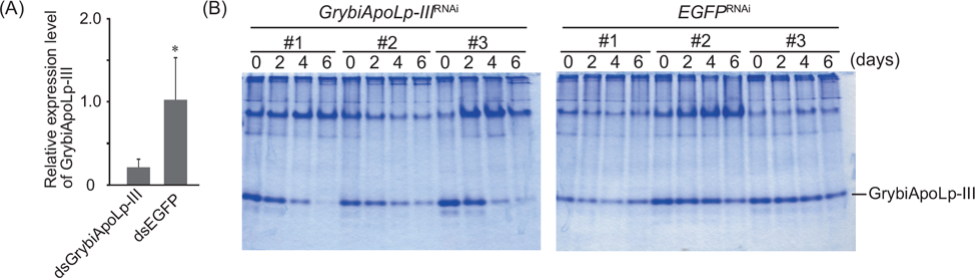
Efficiency of knockdown by *G*. *bimaculatus ApoLp-III*-dsRNA treatment. (A) Quantitative RT-PCR analysis of *GrybiApoLp-III* in *GrybiApoLp-III*-dsRNA-treated (*GrybiApoLp-III*^RNAi^) crickets. RNA was prepared from the fat body of crickets 2 days after dsRNA treatment. *EGFP*-dsRNA was used as an experimental control (*EGFP*^RNAi^). EF (elongation factor) was used as a reference of transcription. Mean + SD (n = 5), *: *P* < 0.01, Student’s-*t* test. (B) Representative data of native-PAGE analyses of hemolymph collected from crickets after *GrybiApoLp-III*-dsRNA treatment (*GrybiApoLp-III*^RNAi^). Hemolymph was collected 0, 2, 4, and 6 days after dsRNA treatment, and was subjected to native-PAGE. *EGFP*-dsRNA was used as an experimental control (*EGFP*^RNAi^). GrybiApoLp-III is indicated by a bar. Numbers represent individual crickets. The reproducibility of this experiment was confirmed by different trials totally using more than 30 individuals from different populations. We also confirmed no off-target effects by dsRNA encoding EGFP using different control gene (DsRed) (S2 Fig).

If ApoLp-III in hemolymph were necessary for the mobilization of lipids in the hemolymph and the maintenance of hemolymph lipid levels, the loss-of-function of ApoLp-III would be expected to change basal hemolymph lipid levels. To test this, we prepared crickets in which *GrybiApoLp-III* transcription was reduced by RNAi. To confirm the effect of RNAi, quantitative RT-PCR was performed using RNA extracted from the fat body of crickets 2 days after receiving *GrybiApoLp-III*-dsRNA or *EGFP*-dsRNA treatments (Fig 3A). Consequent *GrybiApoLp-III* expression was reduced after *GrybiApoLp-III*-dsRNA treatment, whereas it was not altered by *EGFP*-dsRNA treatment. Because ApoLp-III is an abundant protein in the hemolymph, we assessed whether hemolymph GrybiApoLp-III levels were also reduced by the knockdown of *GrybiApoLp-III*. Native PAGE analysis showed that hemolymph ApoLp-III levels gradually declined to undetectable levels by CBB-staining after *GrybiApoLp-III*-dsRNA injection, whereas these levels were not altered by *EGFP*-dsRNA injection (Fig 3B). While the transcriptional reduction by *GrybiApoLp-III*-dsRNA was confirmed from the 2nd day following treatment, the critical effect of *GrybiApoLp-III*-dsRNA on the level of GrybiApoLp-III in the hemolymph was observed from the 4th day following treatment.

### Effect of GrybiApoLp-III knockdown on basal hemolymph lipid levels

**Fig 4:**
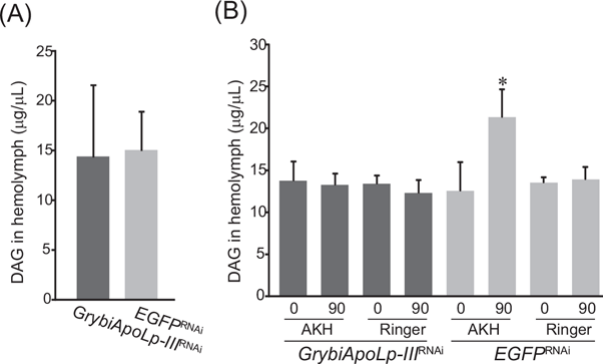
Effect of *G*. *bimacutatus* ApoLp-III knockdown on hemolymph lipid levels. (A) Analysis of basal hemolymph DAG levels in *GrybiApoLp-III*^RNAi^ and *EGFP*^RNAi^ crickets. Values are mean + SD (n = 5). (B) Analysis of basal hemolymph DAG levels in *GrybiApoLp-III*^RNAi^ crickets and hemolymph DAG levels in *GrybiApoLp-III*^RNAi^ crickets after GrybiAKH injection or Ringer’s solution alone. Values are mean + SD (n = 5). Significant differences are denoted by an asterisk (*, *P* < 0.05 by Tukey’s PSD test). Bars without asterisks indicate that differences among levels are not significant.

To elucidate whether GrybiApoLp-III mediates lipid transfer and affects the basal level of hemolymph lipids, we analyzed the effect of GrybiApoLp-III knockdown on hemolymph lipid levels by measuring DAG, a major neutral lipid in the hemolymph. We used crickets 6 days after dsRNA treatment because of the significant reduction in free GrybiApoLp-III levels in the hemolymph (Fig 3B). In contrast to the reduced levels of free GrybiApoLp-III in the hemolymph, basal hemolymph lipid levels did not change in *GrybiApoLp-III*^RNAi^ crickets 6 days after dsRNA treatment as compared to *EGFP*^RNAi^ crickets (Fig 4A).

To confirm the effect of GrybiApoLp-III knockdown on AKH-triggered acute lipid mobilization, we injected a synthetic GrybiAKH peptide into *GrybiApoLp-III*^RNAi^ crickets. This injection resulted in slight changes in the hemolymph lipid levels of *GrybiApoLp-III*^RNAi^ crickets, whereas lipids were significantly mobilized by GrybiAKH injection in *EGFP*^RNAi^ crickets (Fig 4B). Taken together, these results indicate that GrybiApoLp-III is not involved in lipid transfer to maintain basal lipid levels, but is necessary for AKH-triggered acute lipid mobilization.

### Effect of GrybiApoLp-III knockdown on food intake

**Fig 5:**
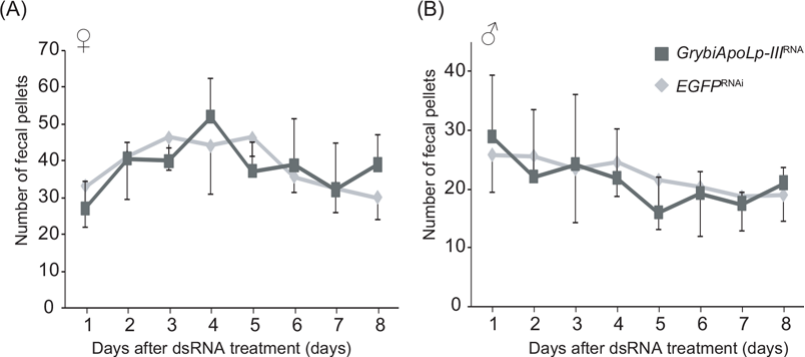
Effect of *G*. *bimaculatus ApoLp-III* knockdown on food intake. Food intake of *GrybiApoLp-III*^RNAi^ adult females (A) and males (B). The amount of food intake was evaluated by counting the number of fecal pellets as previously observed [23]. There were no significant differences between dsGrybiApoLp-III-treated crickets and dsEGFP-treated crickets (*P* > 0.1 by Tukey’s PSD test). Values are mean ± SD (n = 6).

To address whether the effects of *GrybiApoLp-III* knockdown influenced feeding behavior, we analyzed the approximate food intake by adult crickets treated with *GrybiApoLp-III*-dsRNA. We found no difference in the amount of food intake in both female and male *GrybiApoLp-III*^RNAi^ crickets compared to *EGFP*^RNAi^ crickets (Fig 5). These data demonstrate that lowered levels of GrybiApoLp-III in the hemolymph do not influence hemolymph lipid levels and food intake.

### Effect of GrybiLp injection on hemolymph lipid levels and feeding behavior

From analyses using *GrybiApoLp-III*^RNAi^ crickets in which hemolymph lipid levels did not change, we cannot explain how the change in hemolymph lipid levels is linked with feeding behavior, as observed in a previous study using *GrybiAKHR*^RNAi^ crickets [23]. In addition, we did not observe any difference in the amount of food intake by *GrybiApoLp-III*^RNAi^ crickets. To manipulate hemolymph lipid levels, we attempted to inject DAG directly into the hemolymph; however, we could not prepare crickets with increased hemolymph lipid levels (data not shown).

**Fig 6:**
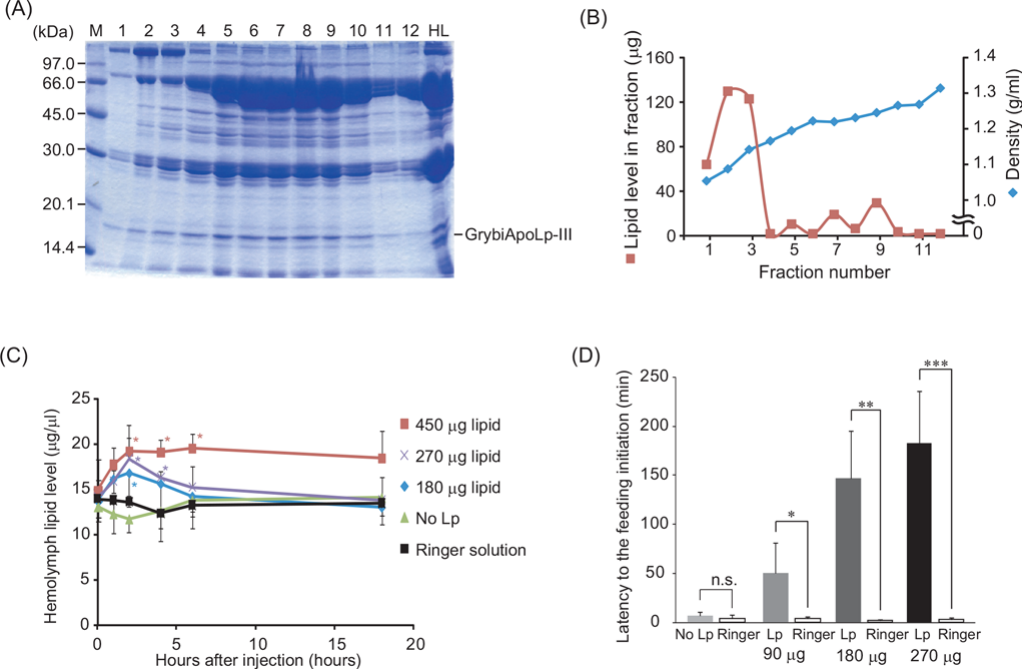
Preparation of Lp and the effect of Lp injection on hemolymph lipid levels and the increased duration to initiate feeding. (A, B) Preparation of GrybiLp. *G*. *bimaculatus* hemolymph was subjected to KBr density gradient ultracentrifugation. (A) After ultracentrifugation, 12 fractions were separated, and the hemolymph before ultracentrifugation was analyzed by SDS-PAGE. (B) Gradients were fractionated from low to high according to density, the specific gravity of each fraction (blue squares). The lipid level of each fraction was also measured (red diamonds). (C) Analysis of hemolymph lipid levels after injection of Lp fractions containing 180, 270, and 450 μg of lipid. Values are mean ± SD (n = 6). Significant differences compared to 0 h are denoted by asterisks (*, *P* < 0.05 by Dunnett’s test). (D) Measurement of duration to the initiation of feeding after injection of Lp fractions containing 90, 180, and 270 μg of lipid. Values are mean + SD (n = 6). Significant differences are denoted by asterisks (*, *P* < 0.05; **, *P* < 0.005; ***, *P* < 0.0005 for Mann-Whitney-test).

Therefore, we injected the lipid-containing Lp fraction, which was purified by ultracentrifugation (Fig 6A and 6B; the purified Lp was confirmed by SDS-PAGE), into the hemolymph to increase hemolymph lipid levels. Injection of Lp fractions containing 180, 270, and 450 μg of lipid resulted in transient, dose-dependent increases in hemolymph lipid levels at 1 to 6 h after injection, whereas injection of the non-Lp-containing fraction had no effect on hemolymph lipid levels (Fig 6C).

Next, to investigate whether Lp injection, which caused increased hemolymph lipid levels, had an effect on feeding behavior, we measured the duration to the initiation of feeding behavior, which is an important index for evaluating feeding motivation in insects [28]. Injection of Lp fractions containing 90, 180, and 270 μg of lipid prolonged the duration to the initiation of feeding in a dose-dependent manner, whereas injection of the non-Lp-containing fraction did not affect this duration (Fig 6D). This result shows that hemolymph lipid levels, when increased above basal levels, inhibit feeding initiation.

## Discussion

In this study, we identified GrybiApoLp-III as a member of the ApoLp-III family by amino acid sequence analysis and cDNA cloning. The similarity of the amino acid sequence between *G*. *bimaculatus* and *A*. *domesticus*, a closely related cricket species, was considerably high (97%). In contrast, the similarity was low (33%) between *G*. *bimaculatus* and a different Orthopteran species, *L*. *migratoria* (S1B Fig) [29]. In general, the similarity of ApoLp-IIIs among other insects is relatively low compared with other conserved proteins [30]. Despite their low similarity, most insect ApoLp-IIIs facilitate lipid mobilization, due to their common structural characteristics and conservation of the predominant expression site in the fat body among insects [20]. Regarding the involvement of GrybiApoLp-III in lipid mobilization, we confirmed that AKH injection and long-term starvation caused a decline in free GrybiApoLp-III levels (Fig 2). This finding indicates that GrybiApoLp-III is used for lipid mobilization and that it would be associated with HDLp to form LDLp for acute lipid mobilization.

**Fig 7:**
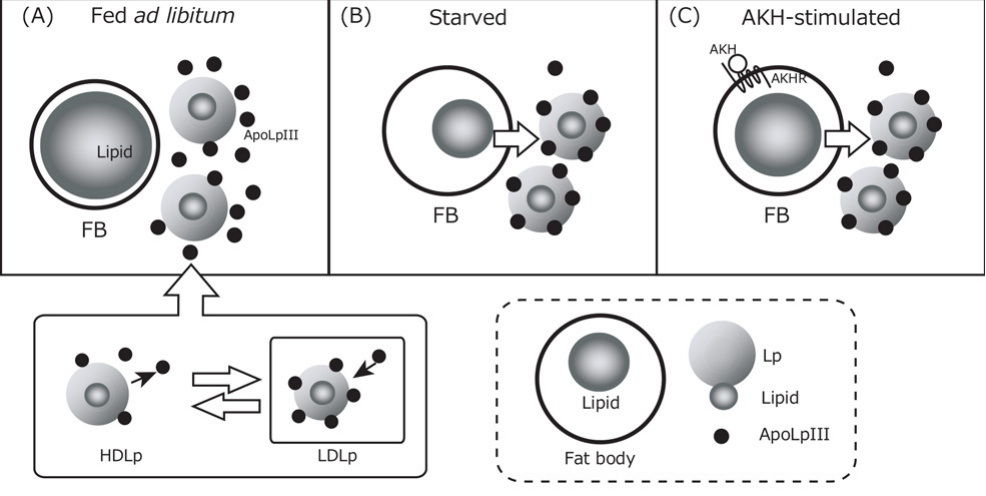
Schematic models to maintain the hemolymph lipid level. (A) For crickets feeding normally, the hemolymph lipid level is maintained by shuttling between HDLp to LDLp; however, the hemolymph lipid level was maintained at similar levels even in GrybiApoLp-III knockdown crickets. (B) For long-term starved crickets, the lowered hemolymph lipid level was recovered from the fat body lipids via LDLp. (C) For AKH-stimulated crickets, possibly under the condition of acute lipid requirements, hemolymph lipid levels increase by lipids of the fat body via LDLp by AKH stimulation.

Knockdown of GrybiApoLp-III could facilitate further analyses of the role of GrybiApoLp-III in the general lipid transfer mechanism, including maintenance of basal hemolymph lipid levels. Interestingly, we found that knockdown of GrybiApoLp-III did not change basal hemolymph lipid levels (Fig 4A). In addition, AKH injection into *GrybiApoLp-III*^RNAi^ crickets had no effect on basal hemolymph lipid levels (Fig 4A). These results show that GrybiApoLp-III is not required by the hemolymph to maintain basal lipid levels during normal feeding (Fig 7A). Alternatively, our results could indicate that other presently unknown factors different from the ApoLp-III-utilizing pathway may be involved in the maintenance of basal hemolymph lipid titers. This result is content with the previous report by Candy et al [31], in which excessive starvation triggered an increase in hemolymph DAG levels but had no effect on AKH levels in the desert locust, *Schistocerca gregaria*. Therefore, the present study would seem to implicate an alternative pathway, which differs from the accepted paradigm for insect lipid mobilization [32]. In contrast, we observed lower levels of free Grybi-ApoLp-III in hemolymph of starved and AKH-stimulated crickets (Fig 2). Together with the data from GrybiApoLp-III knockdown crickets, these results indicate that free GrybiApoLp-III is important for lipid mobilization from the fat body into the hemolymph by forming LDLp under starved and activated AKH signaling conditions (Fig 7B and 7C).

The injection of Lp into the hemolymph led to a transient increase in hemolymph lipid levels that lasted for several hours, while simultaneously decreasing feeding motivation (Fig 6C). We expected that these relatively high hemolymph lipid levels might cause some difference in feeding activity, because we previously demonstrated that lower hemolymph lipid levels, due to AKHR knockdown, resulted in increased food intake [23]. By contrast, we did not observe any changes in food intake even though crickets were treated with GrybiApoLpIII dsRNA (Fig 5), probably because GrybiApoLpIII dsRNA did not influence hemolymph lipid levels (Fig 4A); however, it is intriguing that hemolymph lipid levels were maintained even when crickets fed *ad libitum* did not have ApoLpIII. This implies that inactivation of the lipid-mobilizing capacity by ApoLpIII knockdown did not alter basal hemolymph lipid levels. In addition, the duration to initiate feeding after injection of Lp (Fig 6D) was significantly delayed, suggesting that the hemolymph lipid level is an important factor affecting feeding motivation.

In our previous study, we demonstrated that hemolymph lipid levels decreased and that feeding frequency increased in *GrybiAKHR*^RNAi^ crickets [23]. This finding indicated that hemolymph lipid levels represent a causal factor for motivating feeding behavior in this species. Similarly, in this study, the injection of GrybiLp-containing lipids caused a significant delay to initiate feeding (Fig 6D). This result indicates that increases in hemolymph lipid levels cause a decrease in feeding motivation. Overall, these data strongly indicate that imbalanced hemolymph lipid levels modulate the motivation to feed.

The present study confirms the importance of basal hemolymph nutrient levels. Basal nutrient levels, which are regulated along with certain energy homeostasis pathways, might be archived through mechanisms balancing nutrient uptake and a trade-off between nutrient utilization and storage. Therefore, bias in these mechanisms resulting from imbalanced nutrient levels may alter physiological processes related to feeding behaviors. Based on the results of our study, we conclude that hemolymph lipid levels play a key role in the regulation of feeding behavior by sensing internal nutrient status, which ultimately regulates the frequency of feeding in crickets.

## Supporting Information

S1 Fig cDNA cloning of *GrybiApoLp-III*. (A) A partial cDNA sequence of *GrybiApoLp-III* and its deduced amino acid sequence. Primers used for first the PCR are shown as arrows. Stop codons are marked by an asterisk. Putative polyadenylation sites are underlined. (B) Alignment of GrybiApoLp-III and other ApoLp-III. The alignment of ApoLp-III amino acid sequences was generated using ClustalW and BOXSHADE. The ApoLp-III sequences used in this study are listed in the *Materials and Methods*. (C) Phylogenetic tree of GrybiApoLp-III and other ApoLp-III. The tree was generated by the neighbor-joining method using the amino acid sequences of ApoLp-III. *Homo sapiens* Apolipoprotein-AI was used as an outgroup. The bar represents 0.1 substitutions per site. Numbers represent bootstrap values (%).
(PDF)

S2 Fig Effects of GrybiApoLp-III knockdown using a different control gene, DsRed, from the main text. (A-C) Quantitative RT-PCR analysis of GrybiApoLp-III in GrybiApoLp-III-dsRNA-treated (GrybiApoLp-IIIRNAi) crickets. RNA was prepared from the fat body of crickets 2 days after dsRNA treatment. DsRed-dsRNA was used as an experimental control (DsRedRNAi). Values are mean+SD (n = 6). Significant differences are denoted by asterisks (**, *P* < 0.01; ***, *P* < 0.001 for Student’s *t*-test). (A) EF (elongation factor) was used as a reference of transcription. (B) *rp* (Ribosomal Protein S9) was used as a reference of transcription. (C) *βactin* was used as a reference of transcription. (D) Representative native-PAGE analyses of hemolymph collected from GrybiApoLp-IIIRNAi crickets. Hemolymph was collected 0, 2, 4, and 6 days after dsRNA treatment, and was subjected to native-PAGE. DsRedRNAi crickets were used as an experimental control. GrybiApoLp-III is indicated by a bar. Numbers represent individual crickets. This result was substantially similar to those in the main text, indicating the experimental reproducibility and no off-target effects by control dsRNAs. (E) Analysis of basal hemolymph DAG levels in GrybiApoLp-IIIRNAi and DsRedRNAi crickets. Values are mean + SD (n = 5). Data was statistically analyzed using Student’s *t*-test. (F) Analysis of basal hemolymph DAG levels in GrybiApoLp-IIIRNAi crickets and hemolymph DAG levels in GrybiApoLp-IIIRNAi crickets after GrybiAKH injection or Ringer’s solution alone. Values are mean + SD (n = 5). Significant differences are denoted by asterisks (***, *P* < 0.001 by Tukey’s PSD test). Bars without asterisks indicate that differences among levels are not significant. (G)-(H) Approximate food intake of GrybiApoLp-IIIRNAi adult females (G) and males (H). The approximate amount of food intake was evaluated by counting the number of fecal pellets (23). There were no significant differences between GrybiApoLp-III-treated crickets and DsRed-treated crickets (*P* > 0.1 by Tukey’s PSD test). Values are mean ± SD (n = 6). Primers for qRT-PCR (*rp*) (ACCESSION No. DQ630939) For; 5’-TTTGCAGACCCAAGTGTTCA-3’ Rev; 5’-GCCTTTGGCGAATTAAGACA-3’ Primers for qRT-PCR (*βactin*) (ACCESSION No. AB626808) For; 5’-TTGACAATGGATCCGGAATGT-3’ Rev; 5’-AAAACTGCCCTGGGTGCAT-3’
(EPS)
