# Supplementary material for: Imbalanced Hemolymph Lipid Levels Affect Feeding Motivation in the Two-Spotted Cricket, Gryllus bimaculatus
Source: PLoS One. 2016 May 4;11(5):e0154841. doi: 10.1371/journal.pone.0154841 (PMC4856397; doi:10.1371/journal.pone.0154841)
Supplement: S1 Fig — (A) A partial cDNA sequence of GrybiApoLp-III and its deduced amino acid sequence. Primers used for first the PCR are shown as arrows. Stop codons are marked by an asterisk. Putative polyadenylation sites are underlined. (B) Alignment of GrybiApoLp-III and other ApoLp-III. The alignment of ApoLp-III amino acid sequences was generated using ClustalW and BOXSHADE. The ApoLp-III sequences used in this study are listed in the Materials and Methods. (C) Phylogenetic tree of GrybiApoLp-III and other ApoLp-III. The tree was generated by the neighbor-joining method using the amino acid sequences of ApoLp-III. Homo sapiens Apolipoprotein-AI was used as an outgroup. The bar represents 0.1 substitutions per site. Numbers represent bootstrap values (%). (PDF) [file pone.0154841.s001.pdf]

(A)

```
  1 ACCATCCAGAACGCGCTGCCTTCGCAGGAGGAAGTGCGCACGCAACTGCAGACCCATGCCCAGACCTTTGCCAACAACCTGCAAGCTGCT  90
     T  I  Q  N  A  L  P  S  Q  E  E  V  R  T  Q  L  Q  T  H  A  Q  T  F  A  N  N  L  Q  A  A

 91 GCCACCCAGTTCAACGAGAAGGCCGCCCAGCTGTCCGGCGACGCCCAGACGGCGGTGCGCCAGGCCGCCCAGCACCTCGAGCAGCAAGTG  180
     A  T  Q  F  N  E  K  A  A  Q  L  S  G  D  A  Q  T  A  V  R  Q  A  A  Q  H  L  E  Q  Q  V

181 ACCAACCTGCGCCAGCAGTTCCCCGACGGCGCCCAGGCCGCCGACAAGCTGAAGACGTCCATCGAGAGCGCGCTGGCCGAGGCACGCCGC  270
     T  N  L  R  Q  Q  F  P  D  G  A  Q  A  A  D  K  L  K  T  S  I  E  S  A  L  A  E  A  R  R

271 GTGCAGGAGGCCGTGCAGCCCCACGCAGACGCCGTCGCAGAGTCCCTCAAGACTGCCGCGCGCACCGCCGTCGAGCAGGCTACCGTCATC  360
     V  Q  E  A  V  Q  P  H  A  D  A  V  A  E  S  L  K  T  A  A  R  T  A  V  E  Q  A  T  V  I

361 ACCAACCAGGTGCAGCAGTCTGTGCAGCAGGCCGCCAACGCCCATTAAACGGTGTGTGCAGTGCAGCAGGCCGCCGCCGCCACGCTGACC  450
     T  N  Q  V  Q  Q  S  V  Q  Q  A  A  N  A  H  *

451 TGGCCACCGCTCTCTAGCGGCGGCTGCTCCAGCGGCAGAGAGCGCGCCGACCGCCGCGCCGTACCACGCCACGCGCATGCGCTTGCGACT  540
541 CGCCCCTCGCCCCTCCGCCATCTTGTGTAACTTGGGCGCTAGCGCGGCTGTCGCAGTGTTCTCTGCTATAATATTTTCCAAAGATAATAA  630
631 AGGCGTTTTCTATT(A)n
```

(B)

```
Grybi     1 -----------------------DAPPANAD----FNSLFEAAQRHFQNLTATIQNALPSQ-----EEVRTQLQTHAQTF
Spoex     1 -MVAKLFVLVACIALSHAAMVRRDAPPANTL----LQDIEKHAAEFHKTFSEQLNSIANSK---NTQEVNKAIKEGSDSV
Nillu     1 -MAAKLFVLVACIALSHAAMVRRDAPPANTL----LQDIEKHAAEIHKTFSEQLNSIANSK---NTQEVNKAIKDGSDSV
Trini     1 -MAAKLLVLVACIALSHAGMVRRDAPPS--P----LQDIEKHAAEFQKTFSEQFNSLVNSK---NTQELNKALKDGSDSV
Hypcu     1 -MAAKFIILLALFALSQASVVRRDAPLAN-F----LQDLEKRAADIQKTFSEQFQAISNSK---NVQDVNKAVKESSDVV
Pluxy     1 -------------------MVRREAPAGSTQ----LQDLEKHAQEFQKEFSKQLNSLASSK---NTQELQKALKDGSDSV
Manse     1 MAAKFVVVLAACVALSHSAMVRRDAPAGGNA----FEEMEKHAKEFQKTFSEQFNSLVNSK---NTQDFNKALKDGSDSV
Galme     1 MAAKYVFVVAACSALAQAGIVRRDASTP-------LQDLEKHAAEFQKTFSEQLNAFTNSK---DTKEFNTALKEGSDSV
Bommo     1 -MAAKFVVLFACIALAQGAMVRRDAPDF-------FKDIEHHTKEFHKTLEQQFNSLTKSK---DAQDFSKAWKDGSESV
Bomma     1 -MAAKFIVLFACIALAQGAMVRRDAPDV-------FKDIEHHAKEFQKTFEQQFNSLTKSK---DAQDFSKAWKDGSDSV
Aedae     1 MAKLMFMILAVCLVQVTLARVTREAPAQPEE-NTFFKTLSLIQQKAHDALTGLNQSVLKSLGFQSNDEVVETIQKNTNKY
Culqu     1 MAKLVFIVLALCLVQVSLARVTRDAPAPPAEENQFLKSLSEFGQKFQTALADTQQSVLKALGFQSNEEVVETIQKNTGKY
Anoga     1 MAKLVLIVLALCAVQGSFAMVRRDAPAAPAEEPNFFQSIMSIKDKIEGVFQETQQNVLKSLGFQSNEEVVQTIQTNTNQY
Anosi     1 MAKLVYVVLALCIVQGSFAFVRRDAPVAPVEENNFFKNLLEVGGKIQEAFTETQQSVLKSLGFQSNDEVVQTIQKNTNQY
Achdo     1 MKVIIALVAALACMQVMEARVRRDAGTTGAD----FNSLFEAAQRHFQNLTATIQNALPSQ-----EEVRTQLQTHAQTF
Trica     1 --MAKIFVFVVALIALQGVCAKPKAKQQEKS------TLEELAAQTQVLVNNVTQTLGIKELP-DSKKVVEVLNTNAQNL
Locmi     1 -----MNTLLAVLMLAVAAQARPDAAGH--------VNIAEAVQQLNHTIVNAAHELHETLGLPTPDEALNLLTEQANAF

Grybi    24 ANNLQAAATQFNEKAAQLSGDAQTAVRQAAQHLEQQVTNLRQQF-PDGAQAADKLKTSIESALAEARR-VQEAVQPHADA
Spoex    73 LQQLSALSSSLQNAMTDANAKAKTALEQARQNLEKTAEDLRKAH-PDVERQAGELRNRLQAAVQYTAQEVQKLAKEVASN
Nillu    73 LQQLSALSSSLQSAMTDANAKAKTALEQARQNLEKTAEDLRKSH-PDVERQAGELRTKLQAAVQNTAQEVQKLAKEVASN
Trini    71 LQQLSAFSNSLQGALNDANGKAKTALEEARANLEKTAEELRKAH-PEVEKQAGALREKLQAAVQNTVQETQKLVKEVASN
Hypcu    72 LKQLSTLSSSLQSALTDANGKAKEALEQTRQNLEKTAEELRRAH-PDVEKQANQLRDKLQAAVQSTLQETQKLAKEVAAN
Pluxy    55 LQQISVLSSSLQSALVDANGKAKEALEKTRAELQKTAEQLRAAH-PDVEAKAHELRDTLVAAVQGAPRHSEGLAKEVAAN
Manse    74 LQQLSAFSSSLQGAISDANGKAKEALEQARQNVEKTAEELRKAH-PDVEKEANAFKDKLQAAVQTTVQESQKLAKEVASN
Galme    71 LQQLNALASSLQKALNDANGKAKEALEQTRTNLERTAEELRRAH-PDVERQAGALRDRLQTAVQATVQETQKLAKTVGAN
Bommo    70 LQQLNAFAKSLQGALGDANGKAKEALEQSRQNIERTAEELRKAH-PDVEKNATALREKLQAAVQNTVQESQKLAKKVSSN
Bomma    70 LQQLNAFAKSLQGALGDANGKAKEALEQSRQNIERTAEELRKAH-PDVEKNATALREKLQAAVQNTVQESQKLAKKVSSN
Aedae    80 VEQLKTVQASLDEELKKHSGILDPVVKQLNEKIEETRKSLTEKN-PELVQKAQEYQESVQTRIQSLLTEAQKTGEQLKES
Culqu    81 VDQLKTIQATIQEEAAKHSNIFDPIVKDLNAQIAQTRQKLSEQN-PELVQKAQVYQQTVQANIQSLATEAQKAGERIKEE
Anoga    81 VERLRSVQGVIEEEVKKNSDIFEPILKDLNTKLAQTTATLSEQN-PEVVQKAKEYQAQVQSNVQALVAEAQKTVEKLKED
Anosi    81 VEQLRTVQGTIAEELKKHSDVFEPLVKDLNAKLAETTATLSQQN-PEVVQKAKEYQEQVQANLQSLVSEAQKTVEKLKED
Achdo    72 ANNLQAAATQFNEKAAELSGDAQTAVRQAAQQLEQQVSNLRQQF-PDGAQAADKLKASIESALAEEARRVQEAVQPHADA
Trica    72 ANHVQEIVEKLKTEAKAHQPEVDNVIKQVEQKLSETAATLQQAAGPEATAKAKELKKNLDDGLKTAVAQVEKLVKAVEPD
Locmi    68 KTKIAEVTTSLKQEAEKHQGSVAEQLNAFARNLNNSIHDAATSL------NLQDQLNSLQSALTNVGHQWQDIATKTQAS

Grybi   102 VAES---LKTAARTAVEQATVITNQVQQSVQQAANAH------
Spoex   152 VESTNEKLAPKLREAYENFSKHVEEVQKKVHEAANKQ------
Nillu   152 VEETNEKLAPKLKEAYENFSKHVEEVQKKVHEAASKQ------
Trini   150 VETTNQKLAPKIKEAYDDFVKQAEQVQKKLHEAASKQ------
Hypcu   151 MEQTNEKLAPKIKEAFEDFVKQAEAVQKKVHDAATKQ------
Pluxy   134 LDTANQKLAPKIKEAYEAFAKNAAEVQKKIADAASKQ------
Manse   153 MEETNKKLAPKIKQAYDDFVKHAEEVQKKLHEAATKQ------
Galme   150 LEETNKKLAPQIKSAYDDFVKQAQEVQKKLHEAASKQ------
Bommo   149 VQETNEKLAPKIKAAYDDFAKNTQEVIKKIQEAANAKQ-----
Bomma   149 VQETNEKLAPKIKAAYDDFAKNTQEVIKKIQEAANAKQ-----
Aedae   159 SRGATEQVQTALKQLYDATVDTLQKTVKELEPTKQDQP-----
Culqu   160 GRGASEQLQAALKQLYDVTFQTLQKTTQELEPKKEGSR-----
Anoga   160 TRVPTENIQDALKKLYDYTFETLTKTANELKPKN---------
Anosi   160 TRAPNEELQKALKQIYDSTFETLTKTVNELKPKN---------
Achdo   151 VAES---LKTAARTAVEQATVITNQVQQSVQQAANAH------
Trica   152 ATKAKTDIQNAAQTLLNQIAEVSNNLQAQVKQTIAEHEKTHKN
Locmi   142 AQEAWAPVQSALQEAAEKTKEAAANLQNSIQSAVQKPAN----
```

S1 Fig

(C)

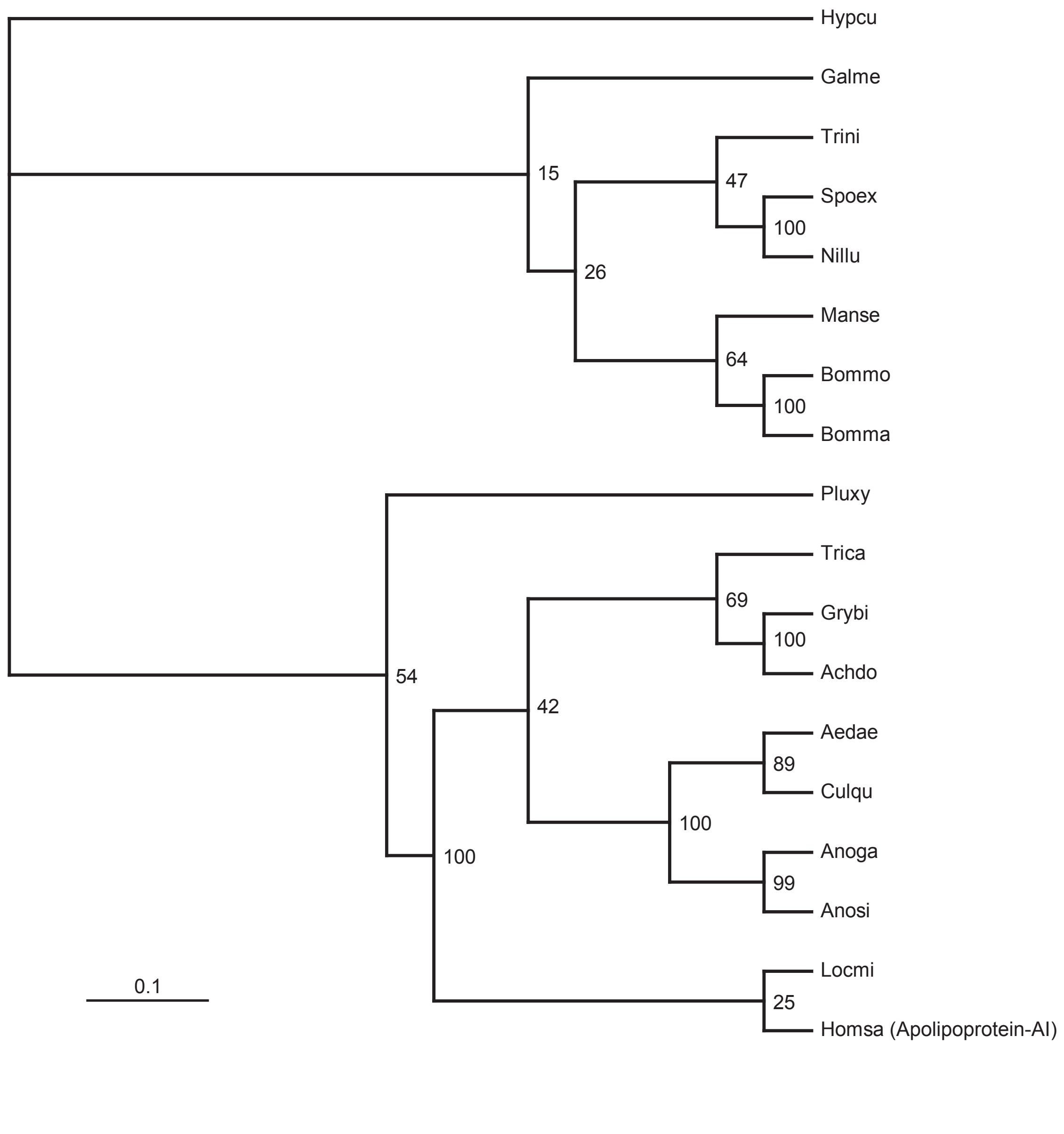
Hypcu
Galme
Trini
Spoex
Nillu
Manse
Bommo
Bomma
Pluxy
Trica
Grybi
Achdo
Aedae
Culqu
Anoga
Anosi
Locmi
Homsa (Apolipoprotein-AI)
15
47
100
26
64
100
54
69
100
42
89
100
100
99
25
0.1

S1 Fig
